# Importance of Autoimmune Responses in Progression of Retinal Degeneration Initiated by Gene Mutations

**DOI:** 10.3389/fmed.2021.672444

**Published:** 2021-12-02

**Authors:** Grazyna Adamus

**Affiliations:** Ocular Immunology Laboratory, Casey Eye Institute, School of Medicine, Oregon Health and Science University, Portland, OR, United States

**Keywords:** autoimmunity, retinal degeneration, inflammation, retinitis pigmentosa, autoantibodies, complement

## Abstract

Inherited retinal diseases (IRDs) are clinically and genetically heterogeneous rare disorders associated with retinal dysfunction and death of retinal photoreceptor cells, leading to blindness. Among the most frequent and severe forms of those retinopathies is retinitis pigmentosa (RP) that affects 1:4,000 individuals worldwide. The genes that have been implicated in RP are associated with the proteins present in photoreceptor cells or retinal pigment epithelium (RPE). Asymmetric presentation or sudden progression in retinal disease suggests that a gene mutation alone might not be responsible for retinal degeneration. Immune responses could directly target the retina or be site effect of immunity as a bystander deterioration. Autoantibodies against retinal autoantigens have been found in RP, which led to a hypothesis that autoimmunity could be responsible for the progression of photoreceptor cell death initiated by a genetic mutation. The other contributory factor to retinal degeneration is inflammation that activates the innate immune mechanisms, such as complement. If autoimmune responses contribute to the progression of retinopathy, this could have an implication on treatment, such as gene replacement therapy. In this review, we provide a perspective on the current role of autoimmunity/immunity in RP pathophysiology.

## Introduction

Inherited retinal diseases (IRDs) are clinically and genetically heterogeneous rare disorders associated with the retinal dysfunction and death of retinal photoreceptor cells. An incidence of IRD is estimated for 1 in 2,000–3,000 individuals, affecting about 2 million people in the world (1). The disease progresses over several decades of patient life and could be a rapid evolution over two decades, or a slow progression that never leads to complete blindness. A dysfunction or death of photoreceptor cells may cause vision loss and blindness. The prognosis of vision loss is difficult to determine because the disease symptoms may depend on a type of inheritance (autosomal dominant, autosomal recessive, or X-linked) and retinal regions involved that includes the periphery, the macula, and both the macula and periphery (2, 3). Furthermore, considering retinal cell contribution to pathology, IRDs can be divided into rod-dominant defect, cone-dominant defect, macular dystrophy, dysfunction of photoreceptors, and bipolar cells, vitreoretinopathies, and hereditary choroidal diseases (3). Among the most frequent and severe forms of those retinopathies is retinitis pigmentosa (RP) which affects 1:4,000 individuals worldwide (1, 4). The objective of this review is to provide a perspective on the current knowledge on the role of autoimmunity/immunity in retinal degeneration initiated by a genetic mutation.

## Retinitis Pigmentosa

The retina consists of two types of photoreceptor cells, rods that are responsible for night vision, and cones for daytime vision and color vision. RP is characterized by degeneration of rods and cones caused initially by the gene mutations, typically affecting rods. Vision loss occurs when the primary rods deteriorate and are eliminated, which usually causes healthy cones to decline next, resulting in blindness (5). The age of RP onset differs and depends on the gene mutations. The rod dysfunction affects the peripheral retina and loss of central vision is the consequence of cone dysfunction, which occurs usually later in life. In addition, when cone degeneration occurs first, it leaves rods mostly unaffected but can cause a severe loss of visual acuity and daylight vision. Early-onset RP is diagnosed when the symptoms of mid-stage RP are already present at 2 years of age and late-onset RP is diagnosed when the symptoms are clinically apparent at or after midlife.

More than 250 genes with about 4,500 causative mutations are identified in different IRD-related diseases (RetNet—Retinal Information Network, http://www.sph.uth.edu/retnet) (6, 7). The genes that have been implicated in syndromic and non-syndromic disease are mostly associated with photoreceptors or RPE, and they involve phototransduction, visual cycle cascade, photoreceptor transcription, and structure (2, 7, 8). Although various genetic mutations have been identified in the patients with RP, the mechanisms by which, these mutations lead to photoreceptor apoptosis, remain mostly unknown (9, 10).

Non-syndromic RP usually involves the peripheral visual field loss, pigment deposits in the fundus, loss of photoreceptor cells as shown at optical coherence tomography (OCT) of the retina, and decreased or absent rod functional responses evaluated by electroretinography (ERG) (11–13). Pigmented deposits, called bone spicules found in the periphery of the retina are a result of photoreceptor cell degeneration. Other classic triads of RP that include intra-retinal pigment migration, optic nerve pallor, and attenuated vessels are not always present on the initial examination. Death of rods can be a direct consequence of genetic mutations; however, death of cones may be caused by the initial death of rods, not to mutations in the cone proteins. Therefore, the period between the onset of rod degeneration and a patient's legal blindness often spans decades (14). To add to the complexity of RP, 20–30% of patients may have an associated non-ocular condition (15).

Most information on the IRD degenerative mechanisms was obtained from the animal models that mimic photoreceptor cell degeneration phenotypes but the knowledge of molecular signaling pathways associated with RP pathogenesis is still incomplete (16, 17). Increasing evidence shows that immune/autoimmune processes may also contribute to the pathogenesis of RP, causing additional retinal degeneration (18). Immune responses could directly target the retina or be a site effect of immunity as a bystander deterioration (19).

The retina has a unique immune defense system, consisting of innate immune cells and the complement system. The sequestration of the eye from the immune system is part of the phenomenon known as an immune privilege (20). Under normal physiological conditions, the retina resides behind the protective blood-retinal barriers, and circulating immune cells are not able to enter the retina (21). In immunologically privileged sites, such as the eye, brain, and testis, autoreactive T cells and B cells can cross from the periphery into the tissue and remain inactive due to the sequestration of antigens behind those barriers (22). However, the sequestration of retinal antigens can be broken by infectious agents or other causes of tissue damage, which may lead to disease development (23). Such an event is dependent on several factors, such as the nature and dose of an antigen, number of exposures, frequency of activated T cells, upregulation of the major histocompatibility complex (MHC), and costimulatory molecules in the affected tissues (24).

## Autoimmunity in Retinal Degeneration

Autoimmunity develops when the immune responses react against the own body, causing inflammation, degeneration, tissue destruction, and organ failure. Autoimmune responses resemble normal immune responses to the pathogens but they are activated by self-antigens or autoantigens. Immune mediated destruction of self-tissue could occur through specific recognition of autoantigens or could be a byproduct of non-specific inflammation (25, 26). Autoimmune diseases have high prevalence (~7–9%) in the population, mostly affecting women, and can cause major illness and death (22). There are different triggers and pathways involved in the pathogenesis of autoimmune diseases (27). The most important feature of an autoimmune disease is the knowledge of an autoantigen involved in the pathogenic process. The retina contains a number of potent autoantigens that are expressed in the thymus and secondary lymphoid tissue, where immunologic tolerance and prevention of autoimmune disease is maintained by a variety of processes, such as clonal deletion and anergy (28–30). Thymic expression is a common feature for all the tissue-specific antigens and their levels of expression play a role in determining the susceptibility to autoimmunity against these molecules.

The other contributory factor to retinal degeneration is inflammation that activates innate immune mechanisms, such as toll-like receptors, inflammasome receptors, and complement components that initiate complex cellular cascades by recognizing or sensing different pathogen and damage-associated molecular patterns (31, 32).

Some observations corroborate that a gene mutation alone might not be responsible for retinal degeneration, e.g., sudden acceleration in photoreceptor decline does not explain degeneration caused by a gene mutation but is an indication that some other processes may be involved. The gene mutations may initiate a stress of photoreceptor cells, secretion of chemokines, and recruitment of microglia to the outer retina, which in effect induces immune (inflammatory cells, cytokines, and chemokines) and autoimmune responses (autoantibodies, autoreactive B cells, and T cells) (33–35). Accumulated microglia secrete cytokines that can cause an increase photoreceptor cell death, disruption of the blood-retina barrier, and attraction of macrophages into the retina (33). Cell death, deposition of debris into subretinal space, and antigens released from dying cells/debris may trigger an autoantibody production (32). The presence of autoantibodies (AAbs) is the consequence of breakdown of tolerance and they are an important serological feature of autoimmune diseases. Initially, circulating AAbs and minor tissue infiltrates may appear without clinical consequences, but later in life, the autoantigens released from the damaged organ may be recognized as foreign substances by the immune system and, in effect, develop pathogenic autoimmunity (autoimmune disease) (19, 26). Altogether, in RP, autoimmunity is likely to be responsible for the progression of photoreceptor cell death that was initiated by a defective gene.

In recent years, a new entity of retinal degenerative disease has been recognized as “autoimmune retinopathy” (AR). AR is often mistaken for RP, because of the overlapping clinical findings and subacute vision loss (36, 37). However, AR has distinctive features that include *progressive* vision loss, often sudden onset later in life, photopsias, and unique visual field defects in the patients without familiar history of RP. In addition, AR is characterized by lack of pigment deposits that often distinguish AR from RP. ARs may present as paraneoplastic syndromes, such as cancer-associated retinopathy and melanoma-associated retinopathy (38–40). Additionally, AR may present without underlying malignancy but have clinical and immunological findings similar to paraneoplastic retinopathies (36, 41, 42). The hallmark of the syndrome are serum AAbs against retinal proteins that may be involved in the pathogenic processes (43–45). Anti-retinal AAbs can persist over the evolution of retinal degeneration and perpetuate the condition (19). Furthermore, cellular immunity is involved in the condition as increased number of memory T cells, NK cells, and decreased regulatory B cell subsets were found in many patients with AR compared with normal controls (37). The role of many different pathways of the immune system in the pathogenesis and progression of AR is under investigation to help with AR diagnosis (46). However, the evidence that the immune system is involved in AR pathogenesis helps with successful treatment of the patients with AR with immunosuppressive drugs, IVIg, and rituximab (42, 47).

## Pathways Contributing to the Death of Photoreceptors

The extent of the immune system activation during RP is still unknown. One can argue that the loss of controlling mechanisms contributes to tissue damage and activation of pro-apoptotic pathways in the retina, ultimately leading to cell death (48–50). To understand its pathology, the immune and autoimmune responses must be examined when a patient first presents some aspects of visual loss. However, the age at onset varies since some patients develop symptomatic visual loss in early childhood, whereas others can remain relatively asymptomatic until mid-adulthood. In addition, the failing photoreceptor cells are phagocytized by microglia to avoid the initiation of inflammation (51, 52). Several studies emphasized that the molecular mechanisms of cell death depends on the caspase-dependent or -independent apoptotic mitochondrial pathway, involving the Bcl-2 family of proteins (53–56). Besides, anti- and pro-apoptotic Bcl-2 protein members exist in retinal cells, suggesting their role in retinal disorders (9, 57). The animal models of retinal degeneration showed that different cell-death pathways could be activated and some of them were genotype-specific (58).

In addition, degeneration of rod photoreceptor cells can be caused by an impairment of autophagy, the process which participates in cell death possibly by initiating apoptosis (59–61). Degradation of proteins by autophagy to prevent the formation of protein aggregates seems to be a necessary process to prevent retinal degeneration (62). Therefore, it is essential to identify all the steps in RP cell death pathways to provide targets for treatment unrelated to the genetic mutations (63). Findings from the animal models have shown that photoreceptor cell death occurred in mice- and rats-expressing mutant rhodopsin in a similar pattern as in humans and the animals manifest clinical signs of autosomal dominant retinitis pigmentosa (ADRP) (64).

The inflammatory cells contribute to retinal degeneration through their cytotoxic effects on photoreceptors (65). Increased levels of pro-inflammatory cytokines and chemokines, in addition to anti-retinal AAbs and immune cells, were detected in sera, aqueous humor, and vitreous of the patients with RP (18, 31, 37, 66, 67) and in the rodent disease models (68, 69). Usually, there is a significant upregulation of the inflammatory markers [interleukin (IL)-1β, IL-6, tumor necrosis factor α (TNF-α), monocyte chemoattractant protein-1 (MCP-1), and ionized calcium binding adaptor molecule 1 (IBA1)] by intraocular cells to start the inflammatory processes (70, 71). In fact, the pro-inflammatory Th1 cytokines (IL-1α, IL-1β, IL-2, IL-6, and INF-γ) characteristic of a cytotoxic response, along with anti-inflammatory Th2 cytokines (IL-4 and IL-10) were found in aqueous humor and vitreous fluid of the patients with RP (31, 66). The vitreous in patients with RP predominantly contained CD4 and CD8 T cells, as well as human leukocyte antigen (HLA)-DR activated cells and some B cells. Moreover, serum high-sensitivity C-reactive protein (hs-CRP) was significantly increased in the patients with RP, and higher hs-CRP was associated with faster deterioration of central visual function (72). The patients with an increased number of inflammatory cells showed reduced visual function (reduced visual acuity and visual fields). All those factors may contribute to the progression of the retinal degeneration, and systemic and local inflammation can change overtime with the progression of tissue degeneration in RP (31, 37).

## Autoantibodies in Retinitis Pigmentosa

Autoantibodies (AAbs) are frequently found in RP and in healthy individuals. High-affinity pathogenic AAbs are produced by antigen-stimulated B cells that undergo somatic hypermutation to become long-lived plasma cells as a result of the self-tolerance breakdown (73, 74). Serum IgG autoantibody profiles are unique to an individual and may be remarkably stable over time (75). Presence of circulating AAbs specific for photoreceptor antigens raises the possibility of their pathogenic role (19).

In recent years, significant progress has been made in understanding the role of anti-retinal AAbs in pathogenesis, diagnosis, and management of AR, such as paraneoplastic retinopathies (45, 76–78). Since the ocular findings in ARs are similar to those found in many forms of RP, especially those that do not have family history of retinal degeneration, one could hypothesize that an underlying autoimmunity could cause, or at least contribute to, the progression of retinal disease. In early studies, the high levels of anti-retinal IgG and IgM antibodies were found in various cohorts of the patients with RP (79–81). However, specificities of those AAbs have not been determined by the investigators. Later studies showed ~2% sera of simplex patients with RP possessed anti-recoverin AAbs, which let the authors to hypothesis that anti-recoverin AAbs exacerbate the underlying RP disease (76). This is in an agreement with the recent study that showed the patients with RP over 50 years old with identified gene mutation and history of cancer, had serum anti-recoverin AAbs (82). Such AAbs occurred more likely in the patients with RP with cancer than in the patients without cancer. This suggests that anti-recoverin AAbs were generated in response to cancer rather than to degenerating retina due to the gene mutation, because the mutations in the tumor genome can cause tumors to express mutant proteins, such as recoverin, that is normally expressed on the retina. Moreover, anti-retinal AAbs were reported secondary to the gene defects in the patients with RP (18, 66, 72) but their role have not been fully explained.

### Autoantibody Targets Are the Same Proteins as Mutated Gene Products in RP

The likely sequence of events in the generation of anti-retinal AAbs in RP is the death of photoreceptor cells induced by a gene mutation, which causes the release of antigenic proteins that are then captured by the potential antigen-presenting cells (e.g., macrophages), and breakdown of the blood-retinal barrier during that process (83). An abnormal gene may lead to an abnormal protein or an abnormal amount of a normal protein, and mutated proteins can cause pathology by misfolding and aggregation. Those proteins can be targets of the autoimmune response, especially when mutation leads to photoreceptor degeneration (69). When photoreceptors die, it would be expected that the immune system targets freed proteins from failing outer segments and elicits AAbs against those autoantigens with the help of macrophages. Some patients with RP may have serum AAbs against retinal proteins that were subject to disease-causing mutation (8, 39). For example, AAbs against arrestin were detected in the patients with RP as well in the patients with autoimmune uveitis or autoimmune retinopathy (84, 85). However, the degree of immune reactivity against arrestin and the severity of disease in the patients with RP are not strongly correlated. This observation suggests that the immune responses to the retinal autoantigens are regulated by factors other than the level of retinal damage and the release of antigens from the affected tissues. The systemic autoimmune responses may play a bigger role in formation of AAbs.

The presence of AAbs in RP led to a hypothesis that autoimmunity could be responsible for the progression of photoreceptor cell death initiated by a genetic mutation. A majority of causative mutations in RP involve proteins that participate in the phototransduction cascade, such as rhodopsin (RHO), the catalytic unit and subunits of PDE6 (PDE6A and PDE6B, respectively), the subunit of the rod cyclic nucleotide gated channel (CNGA1), and arrestin (SAG) (86). The patients with AR have AAbs against phototransduction proteins (77). Detection of anti-retinal AAbs suggests a generation of AAbs started against mutant proteins in RP. However, whether AAbs are made to a wild-type protein or mutant protein has yet to be determined. The explanation of the role of specific mutations as etiological causes for RP must mostly depend on their ability to induce the pathogenic mutant proteins that cause structural and functional changes in the cell, leading to retinal pathology (87).

Recent studies on neurodegeneration in multiple sclerosis (MS) showed that both, mutant and wild myelin protein PLP1 were able to generate the immune responses (88). Using wild type and mutant peptide microarrays, several serum AAbs against multiple mutated PLP1 have been found in those patients. Anti-mutant PLP1 autoantibody responses provided evidence that PLP1 mutations conceivably elicit the immune-mediated destruction of myelin (88). We postulate that the retinal proteins altered by a gene mutation in RP, act as new autoantigens, thus AAbs may be generated with similar specificities as to native proteins. It is not easy to determine whether the patients have autoimmunity to a native or mutant protein. Explaining the specific role of mutations as etiological factors for RP relies on their ability to induce the structural changes in proteins that have pathophysiological consequences (87). Changes in the net charge of a protein may lead to conformational modifications in the tertiary and quaternary structure of that protein, and alters the interaction with other proteins, especially human HLA molecules. This would apply only to the mutations that change the amino acid sequences in such a way that influence the structure and function of proteins (89). The mutant-proteins accumulate during retinal degeneration and can be seen by the immune system as a new and amplify the autoimmune response, eventually leading to autoimmune pathology. Also, the posttranslational modifications, such as a protein citrullination can trigger the activation of the immune system, both locally and systemically for AAbs production, contributing to disease pathogenesis in RP (35, 90, 91). These findings suggest that the presence of mutations and associated immune response could be part of the pathogenesis of RP.

Few years ago, it has been proposed that the genes encoding for the proteins that become autoantigens could have a fundamental propensity toward mutation (92). According to the study, the autoantigens contain significantly more single nucleotide polymorphisms (SNP) than other human genes do. The SNPs may represent an essential requirement for the primary generation of an autoimmune response. Structural features of a given autoantigen can be prerequisite to determine whether such an antigen is suitable to induce autoimmune response (89). Thus, the autoantibody repertoire to the retinal antigens is represented by pro-inflammatory and immunological properties of autoantigens (93, 94). The ability of new antigens released from the damaged cells and tissues may act as chemoattractants for leukocytes, which is an important step in promoting inflammation and favoring the development of autoimmunity (93). In fact, two retina-specific proteins, arrestin and interphotoreceptor retinoid-binding protein (IRBP) were found to be chemoattractants for lymphocytes and immature dendritic cells (95). These autoantigens, which have no primary or secondary structural homology to chemokines, induce cell migration by interacting with specific chemokine receptors. IRBP interacts with chemokine receptors CXCR5 and CXCR3, and arrestin interacts with CXCR3, and both the proteins can facilitate retinal damage by inflammatory and immune responses, and potentially contribute to the development of autoimmune diseases, such as autoimmune uveitis (95). Moreover, during the course of disease, specific AAbs bind stronger with the target antigens in the later stage than those occurring in the beginning (96).

### Association Between Cystoid Macular Edema With Anti-retinal Autoantibodies

The patients with RP experience central vision loss in the form of cystoid macular edema (CME), which can form at any stage of RP, in one or both the eyes, and in any genetic form but is more often associated with Crumbs homolog (cell polarity complex component) (CRB1) mutations (97). The origin of macular edema remains poorly understood. CME is a major cause of vision loss in uveitis (98). Anti-retinal AAbs, vitreous traction, retinal pigment epithelium dysfunction, and Müller cell edema can contribute to the pathology of CME (97). AAbs against two enzymes, carbonic anhydrase II and enolase were detected in the patients with bilateral CME and RP, suggesting that these two enzymes play an important role in foveal function (99). The high prevalence of anti-CAII and anti-enolase AAbs in the patients with CME have also been found in a German group of patients with CME (100). The authors proposed that blocking of CAII and enolase activity by AAbs in the RPE may be a major cause of edema formation. Independently, our laboratory has also found the presence of anti-CAII AAbs in the patients with PR with CME, further corroborating their role in pathology of edema (101). In addition, the higher levels of intraocular cytokines, such as IL-2 have been found in the patients with CME, impaling their role of inflammation (66). This suggests that inflammatory mediators as well as AAbs may contribute to the development of inflammatory CME, but the exact mechanism for the CME development and its persistence is still unknown.

## HLA and Retinitis Pigmentosa

A strong association between the HLA region and autoimmune disease has been established over 50 years. The HLA molecules are responsible for the induction and regulation of immune responses, and selection of T cell repertoire (102). The class II molecules, such as HLA-DR, DP, and DQ present exogenous peptides that are expressed on antigen-presenting cells (dendritic cells, macrophages, and B cells) and activated T cells. The likely mechanisms, by which HLA polymorphisms could contribute to the development of RP, may be related to the presentation of autoantigens, the shared epitope, and molecular mimicry. The only studies of HLA association and RP were performed over 30 years ago (103). HLA serological typing study of 173 patients with autosomal dominant and recessive RP was not different than the frequency of HLA antigens in control population (103). Then, the study of 10 patients with autosomal recessive RP showed a significant increase in the frequency of the antigens Cw4, Cw6, and DR11 (104). In other retinal diseases, the patients with severe diabetic retinopathy had frequent alleles on the DR3-DQ2 haplotype, such as DRB1*0301, DQA1*0501, and DQB1*0201 (105). The association between acute retinal necrosis syndrome and certain HLA specificities suggested immune predisposition to the disease (106). RPE cells phagocytose and recycle autoantigen-rich retinal rod outer segments and co-express HLA DR and DQ Class II antigens in response to IFN-gamma stimulation (107). This suggests that the RPE cells may play an immunoregulatory function in autoimmunity to the retinal antigens as primary inducers and/or as suppressors of retinal inflammation (108). Further studies are needed to understand whether the HLA polymorphism influence the development of RP.

## Innate Responses—Contributory Factor?

There is some evidence that chronic inflammation is associated with the pathogenesis of RP (109, 110). The indications of chronic inflammation in the patients with RP and the rodent models include the presence of serum retinal AAbs, immune cells in the vitreous cavity of affected individuals, and increased levels of pro-inflammatory cytokines and chemokines in aqueous humor and vitreous fluid of the patients with RP (31, 32).

The retina has a unique immune defense system, consisting of innate immune cells and the complement system. Microglia that includes microglia (resident macrophages), perivascular macrophages, and dendritic cells play an important role in the retinal immune defense (111, 112). They are located behind the blood-retina barriers within an immune-privileged microenvironment in the inner layers of the retina, such as the ganglion layer, inner plexiform layer, and outer plexiform layer (113, 114).

Under normal physiological conditions, microglia are resting but in the disease state, the activated microglia change their shape and perform several important functions in the retina that includes phagocytosis of debris and apoptotic cells, maintenance of synapses, and response to inflammation (114). Phagocytosis may actively induce apoptosis and those apoptotic photoreceptors are selectively eliminated from the outer nuclear layer to the subretinal space, and then phagocytosed by monocyte-derived macrophages (115). The activation of microglia contributes to retinal damage and disease progression (69, 116, 117). Microglia have different functions depending on the underlying cause of retinal degeneration (118, 119). In RP, the death of rod photoreceptors may attract resident microglia that become activated, depending on the local and systemic cytokines secretion, then migrate to the outer retina to phagocytose rod cell debris from dying cells (1, 48). Infiltrating microglia secrete pro-inflammatory cytokines that stimulate photoreceptor apoptosis (34, 63). Increased secretion of TNF-α and IL-1b was found shortly after disease onset (120). The studies from our laboratory, examining the evolution of autoimmune responses against retina in naive dystrophic RCS rats over the course of their retinal degeneration, linked the occurrence of anti-retinal autoantibodies to the entry of activated macrophage/microglia, suggesting their role in neurodegeneration (69). Microglial activation is independent of the underlying genetic defect, and it is not a side effect of hereditary photoreceptor dystrophies, but can arise by the availability of endogenous retinal proteins from the dying photoreceptors (121).

In addition, microglia are the source of complement and complement-regulatory factors that are markedly up-regulated in the human retinas with RP (122). The complement system has an integral role in maintaining immune surveillance and homeostasis in the eye microenvironment but overstimulation of the complement system can induce retinal pathology and ocular inflammation (32, 122). Complement mediates a wide range of functions in the tissue and can be activated by three distinct pathways: classical, alternative, and lectin. The studies using the animal models of RP showed an involvement of complement proteins in retinal degeneration (123). For example, in the rd10 mouse that is caused by a spontaneous mutation in Pde6β gene, at the stage when rod photoreceptors have completely degenerated, there was an increase in many classical and alternative complement pathway components, such as C1q, C1r, C3, and C4 (124). However, photoreceptor degeneration in the rd1 mouse with a naturally occurring null mutation within the gene encoding Pdeβ was unaffected by C1q component (125). In contrast, the levels of C1q progressively increased over the course of photoreceptor degeneration in the Rho–/– mouse when the mice lost all the rods over 3-month period by apoptosis. The C3 and its receptor CR3 signaling regulate the microglia–photoreceptor interactions. The deficiency of C3–CR3 lead to decrease microglial phagocytosis of apoptotic photoreceptors and increase microglial neurotoxicity to photoreceptor cells in RP (123). Another complement protein C1q is shown to be the primary component of cone photoreceptor survival factor (126). In the normal adult RPE–choroid, the choroidal cells are the predominant local source of most alternative complement pathway components and regulators (127). Moreover, the occurrence of reactive complement proteins on the surface of RPE cells may accelerate lipofuscin accumulation by inhibiting their clearance (128). These findings have potential implications for the pathological mechanisms independent of genetic mutation and new targets for therapy of retinal degeneration. Targeting the microglia (e.g., minocycline) may reduce the production of several pro-inflammatory mediators thus may result in broader beneficial effects than just inhibition of single cytokines (129).

## Final Remarks

**Figure 1:**
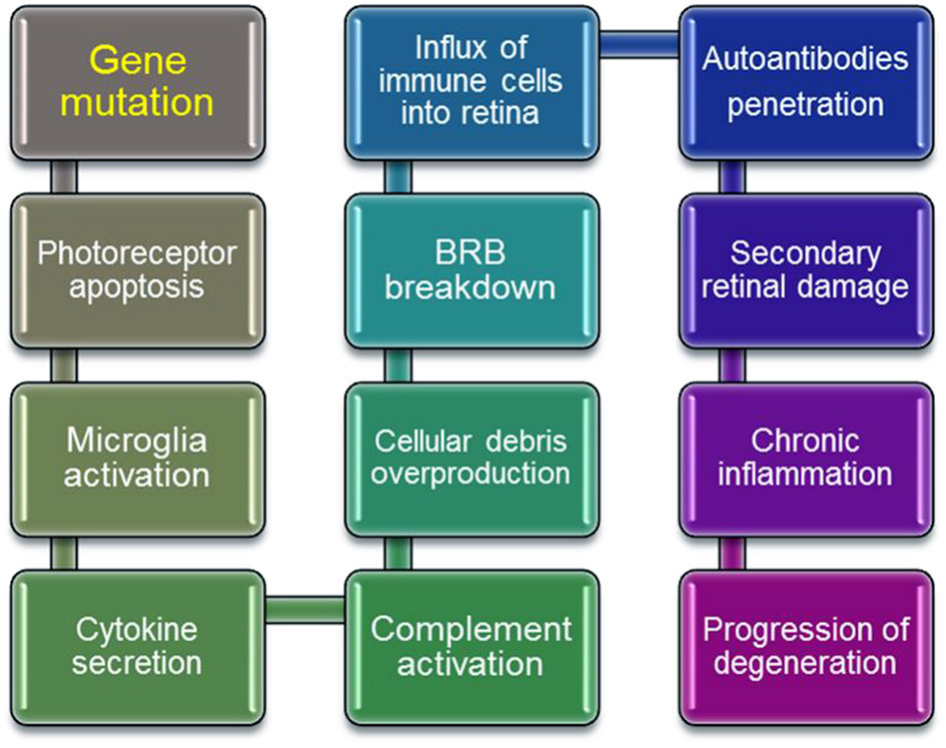
Schematic steps in progressive retinal degeneration. Mutant-proteins accumulate during retinal degeneration and can be seen as new, which can amplify the autoimmune response, ultimately leading to autoimmune pathology. Activation of immune cells by overproduction of cellular debris due to photoreceptor death results in the inner Blood Retina Barrier (BRB) breakdown, which invites systemic macrophages into the retina. Resident and circulating macrophages can contribute to secondary retinal damage from inflammation, and in effect ameliorate retinal degeneration.

Inherited retinal diseases represent a highly heterogeneous group of disorders that have one common element: abnormal visual function originating at the death of retinal photoreceptors. The gene defects can initiate death of retinal cells that can progress further to symptomatic changes mediated by immune and autoimmune responses (Figure 1). An initial gene mutation followed by sudden loss and progressive nature of retinal degeneration suggests the involvement of autoimmune responses. Since there are a variety of genes and mutations that cause retinal degeneration, gene replacement therapy approaches that are currently in development may be time-consuming and cost-prohibitive for treatment of all forms of RP. If the autoimmune responses contribute to the progression of retinopathy this could have implication on development of retinal degeneration and success of gene replacement therapy. Alternative approaches can be based on the immunological pathways that cause retinal degeneration in different forms of RP. In such cases, immunomodulatory and biologic drugs targeting B cells could be beneficial in slowing retinal degeneration caused by a gene mutation. More studies are needed to fully establish the role of autoimmunity in different forms of retinal degenerations.

## Author Contributions

The sole author is responsible for the design and writing of the review.

## Funding

This study was supported by grant P30 EY010572 from the National Institutes of Health (Bethesda, MD, USA) and by unrestricted departmental funding from the Research to Prevent Blindness (New York, NY, USA).

## Conflict of Interest

The author declares that the research was conducted in the absence of any commercial or financial relationships that could be construed as a potential conflict of interest.

## Publisher's Note

All claims expressed in this article are solely those of the authors and do not necessarily represent those of their affiliated organizations, or those of the publisher, the editors and the reviewers. Any product that may be evaluated in this article, or claim that may be made by its manufacturer, is not guaranteed or endorsed by the publisher.

## References

[1] Hartong DT Berson EL Dryja TP. Retinitis pigmentosa. Lancet. (2006) 368:1795–809. 10.1016/S0140-6736(06)69740-7 17113430

[2] Wang DY Chan WM Tam POS Baum L Lam DSC Chong KKL. Gene mutations in retinitis pigmentosa and their clinical implications. Clinica Chimica Acta. (2005) 351:5–16. 10.1016/j.cccn.2004.08.004 15563868

[3] Hohman TC. Hereditary retinal dystrophy. In: Whitcup SM Azar DT editors, Pharmacologic Therapy of Ocular Disease. Cham: Springer International Publishing (2017). p. 337–67. 10.1007/164_2016_91

[4] Hamel C. Retinitis pigmentosa. Orphanet J Rare Dis. (2006) 1:40. 10.1186/1750-1172-1-40 17032466 PMC1621055

[5] Campochiaro PA Mir TA. The mechanism of cone cell death in Retinitis Pigmentosa. Prog Retin Eye Res. (2018) 62:24–37. 10.1016/j.preteyeres.2017.08.004 28962928

[6] Sorrentino FS Gallenga CE Bonifazzi C Perri PA. Challenge to the striking genotypic heterogeneity of retinitis pigmentosa: a better understanding of the pathophysiology using the newest genetic strategies *Eye*. (2016) 30:1542–8. 10.1038/eye.2016.197 27564722 PMC5177762

[7] Farrar GJ Carrigan M Dockery A Millington-Ward S Palfi A Chadderton N. Toward an elucidation of the molecular genetics of inherited retinal degenerations. Hum Mol Genet. (2017) 26:R2–11. 10.1093/hmg/ddx185 28510639 PMC5886474

[8] Birtel J Gliem M Mangold E Muller PL Holz FG Neuhaus C. Next-generation sequencing identifies unexpected genotype-phenotype correlations in patients with retinitis pigmentosa. PLoS ONE. (2018) 13:e0207958. 10.1371/journal.pone.0207958 30543658 PMC6292620

[9] Sancho-Pelluz J Arango-Gonzalez B Kustermann S Romero F van Veen T Zrenner E. Photoreceptor cell death mechanisms in inherited retinal degeneration. Mol Neurobiol. (2008) 38:253–69. 10.1007/s12035-008-8045-9 18982459

[10] Ferrari S Di Iorio E Barbaro V Ponzin D Sorrentino FS Parmeggiani F. Retinitis pigmentosa: genes and disease mechanisms. Curr Genomics. (2011) 12:238–49. 10.2174/138920211795860107 22131869 PMC3131731

[11] Verbakel SK van Huet RAC Boon CJF den Hollander AI Collin RWJ Klaver CCW. Non-syndromic retinitis pigmentosa. Progr Retinal Eye Res. (2018) 3:5. 10.1016/j.preteyeres.2018.03.005 29597005

[12] Granse L Ponjavic V Andreasson S. Full-field ERG, multifocal ERG and multifocal VEP in patients with retinitis pigmentosa and residual central visual fields. Acta Ophthalmol Scand. (2004) 82:701–6. 10.1111/j.1600-0420.2004.00362.x 15606467

[13] Birch DG Anderson JL Fish GE. Yearly rates of rod and cone functional loss in retinitis pigmentosa and cone-rod dystrophy. Ophthalmology. (1999) 106:258–68. 10.1016/S0161-6420(99)90064-7 9951474

[14] Lobanova ES Finkelstein S Li J Travis AM Hao Y Klingeborn M. Increased proteasomal activity supports photoreceptor survival in inherited retinal degeneration. Nat Commun. (2018) 9:1738. 10.1038/s41467-018-04117-8 29712894 PMC5928105

[15] Werdich XQ Place EM Pierce EA. Systemic diseases associated with retinal dystrophies. Semin Ophthalmol. (2014) 29:319–28. 10.3109/08820538.2014.959202 25325857

[16] Collin GB Gogna N Chang B Damkham N Pinkney J Hyde LF. Mouse models of inherited retinal degeneration with photoreceptor cell loss. Cells. (2020) 9:931. 10.3390/cells9040931 32290105 PMC7227028

[17] Newton F Megaw R. Mechanisms of photoreceptor death in retinitis pigmentosa. Genes. (2020) 11:1120. 10.3390/genes11101120 32987769 PMC7598671

[18] McMurtrey JJ Tso MOM. A review of the immunologic findings observed in retinitis pigmentosa. Survey Ophthalmol. (2018) XX:1–13. 10.1016/j.survophthal.2018.03.002 29551596

[19] Adamus G. Are anti-retinal autoantibodies a cause or a consequence of retinal degeneration in autoimmune retinopathies? Front Immunol. (2018) 9:765. 10.3389/fimmu.2018.00765 29713325 PMC5911469

[20] Taylor AW. Ocular immune privilege. Eye. (2009) 23:1885–9. 10.1038/eye.2008.382 19136922 PMC4698145

[21] Greenwood J. Mechanisms of blood-brain barrier breakdown. Neuroradiology. (1991) 33:95–100. 10.1007/BF00588242 2046916

[22] Theofilopoulos AN Kono DH Baccala R. The multiple pathways to autoimmunity. Nat Immunol. (2017) 18:716–24. 10.1038/ni.3731 28632714 PMC5791156

[23] Stein-Streilein J Caspi RR. Immune privilege and the philosophy of immunology. Front Immunol. (2014) 5:110. 10.3389/fimmu.2014.00110 24678312 PMC3959614

[24] Gery I Caspi RR. Tolerance induction in relation to the eye. Front Immunol. (2018) 9:2304. 10.3389/fimmu.2018.02304 30356688 PMC6189330

[25] Forrester JV Kuffova L Dick AD. Autoimmunity, autoinflammation and infection in uveitis. Am J Ophthalmol. (2018) 2:19. 10.1016/j.ajo.2018.02.019 29505775

[26] Lleo A Invernizzi P Gao B Podda M Gershwin ME. Definition of human autoimmunity - autoantibodies versus autoimmune disease. Autoimmunity Rev. (2010) 9:A259–66. 10.1016/j.autrev.2009.12.002 19963079

[27] Lifeng W Fu-Sheng W Eric GM. Human autoimmune diseases: a comprehensive update. J Internal Med. (2015) 278:369–95. 10.1111/joim.12395 26212387

[28] Egwuagu CE Charukamnoetkanok P Gery I. Thymic expression of autoantigens correlates with resistance to autoimmune disease. J Immunol. (1997) 159:3109–12. 9317106

[29] Charukamnoetkanok P Fukushima A Whitcup SM Gery I Egwuagu CE. Expression of ocular autoantigens in the mouse thymus. Curr Eye Res. (1998) 17:788–92. 10.1080/02713689808951259 9723993

[30] Voigt V Wikstrom ME Kezic JM Schuster IS Fleming P Makinen K. Ocular antigen does not cause disease unless presented in the context of inflammation. Sci Rep. (2017) 7:14226. 10.1038/s41598-017-14618-z 29079770 PMC5660195

[31] Yoshida N Ikeda Y Notomi S Ishikawa K Murakami Y Hisatomi T. Clinical evidence of sustained chronic inflammatory reaction in retinitis pigmentosa. Ophthalmology. (2013) 120:100–5. 10.1016/j.ophtha.2012.07.006 22986109

[32] Sudharsan R Beiting DP Aguirre GD Beltran WA. Involvement of innate immune system in late stages of inherited photoreceptor degeneration. Sci Rep. (2017) 7:17897. 10.1038/s41598-017-18236-7 29263354 PMC5738376

[33] Cuenca N Fernández-Sánchez L Campello L Maneu V De la Villa P Lax P. Cellular responses following retinal injuries and therapeutic approaches for neurodegenerative diseases. Prog Ret Eye Res. (2014) 43:17–75. 10.1016/j.preteyeres.2014.07.001 25038518

[34] Zhao L Zabel MK Wang X Ma W Shah P Fariss RN. Microglial phagocytosis of living photoreceptors contributes to inherited retinal degeneration. EMBO Molec Med. (2015) 7:1179–97. 10.15252/emmm.201505298 26139610 PMC4568951

[35] Hollingsworth TJ Gross AK. Innate and autoimmunity in the pathogenesis of inherited retinal dystrophy. Cells. (2020) 9:630. 10.3390/cells9030630 32151065 PMC7140441

[36] Heckenlively J Ferreyra H. Autoimmune retinopathy: a review and summary. Semin Immunopathol. (2008) 30:127–34. 10.1007/s00281-008-0114-7 18408929

[37] Heckenlively JR Lundy SK. Autoimmune retinopathy: an immunologic cellular-driven disorder. In: Ash J Anderson R LaVail M Bowes Rickman C Hollyfield J Grimm C editors, Retinal Degenerative Diseases. Advances in Experimental Medicine and Biology. Cham: Springer International Publishing (2018). p. 193–201. 10.1007/978-3-319-75402-4_24 29721944

[38] Thirkill CE Roth AM Keltner JL. Cancer-associated retinopathy. Archiv Ophthalmol. (1987) 105:372–5. 10.1001/archopht.1987.01060030092033 2950846

[39] Adamus G. Paraneoplastic retinal degeneration. In: Levin L Albert DM editor, Ocular Disease: Mechanisms and Management. Saunders Elsevier, Inc. (2010). p. 599–608. 10.1016/B978-0-7020-2983-7.00076-0

[40] Lu Y Jia L He S Hurley MC Leys MJ Jayasundera T. Melanoma-associated retinopathy: a paraneoplastic autoimmune complication. Arch Ophthalmol. (2009) 127:1572–80. 10.1001/archophthalmol.2009.311 20008709 PMC4618318

[41] Mizener JB Kimura AE Adamus G Thirkill CE Goeken JA Kardon RH. Autoimmune retinopathy in the absence of cancer. Am J Ophthalmol. (1997) 123:607–18. 10.1016/S0002-9394(14)71073-6 9152066

[42] Fox AR Gordon LK Heckenlively JR Davis JL Goldstein DA Lowder CY. Consensus on the diagnosis and management of nonparaneoplastic autoimmune retinopathy using a modified delphi approach. Am J Ophthalmol. (2016) 168:183–90. 10.1016/j.ajo.2016.05.013 27210277 PMC4969197

[43] Adamus G Ren G Weleber RG. Autoantibodies against retinal proteins in paraneoplastic and autoimmune retinopathy. BMC Ophthalmol. (2004) 4:5. 10.1186/1471-2415-4-5 15180904 PMC446200

[44] Adamus G. Latest updates on antiretinal autoantibodies associated with vision loss and breast cancer. Invest Ophthalmol Vis Sci. (2015) 56:1680–8. 10.1167/iovs.14-15739 25754855 PMC4354244

[45] Adamus G Champaigne R Yang S. Occurrence of major anti-retinal autoantibodies associated with paraneoplastic autoimmune retinopathy. Clin Immunol. (2020) 210:108317. 10.1016/j.clim.2019.108317 31770612 PMC6989367

[46] Sobrin L. Progress toward precisely diagnosing autoimmune retinopathy. Am J Ophthalmol. (2018) 188:xiv-xv. 10.1016/j.ajo.2018.01.002 29396036

[47] Davoudi S Ebrahimiadib N Yasa C Sevgi DD Roohipoor R Papavasilieou E. Outcomes in autoimmune retinopathy patients treated with rituximab. Am J Ophthalmol. (2017) 180:124–32. 10.1016/j.ajo.2017.04.019 28483493

[48] Gupta N Brown KE Milam AH. Activated microglia in human retinitis pigmentosa, late-onset retinal degeneration, and age-related macular degeneration. Exp Eye Res. (2003) 76:463–71. 10.1016/S0014-4835(02)00332-9 12634111

[49] Karlstetter M Ebert S Langmann T. Microglia in the healthy and degenerating retina: Insights from novel mouse models. Immunobiology. (2010) 215:685–691. 10.1016/j.imbio.2010.05.010 20573418

[50] Noailles A Maneu V Campello L Gómez-Vicente V Lax P Cuenca N. Persistent inflammatory state after photoreceptor loss in an animal model of retinal degeneration. Sci Rep. (2016) 6:33356. 10.1038/srep33356 27624537 PMC5022039

[51] Nagata S Hanayama R Kawane K. Autoimmunity and the clearance of dead cells. Cell. (2010) 140:619–30. 10.1016/j.cell.2010.02.014 20211132

[52] Okunuki Y Mukai R Pearsall EA Klokman G Husain D Park DH. Microglia inhibit photoreceptor cell death and regulate immune cell infiltration in response to retinal detachment. Proc Natl Acad Sci USA. (2018) 115:E6264–73. 10.1073/pnas.1719601115 29915052 PMC6142210

[53] Portera-Cailliau C Sung CH Nathans J Adler R. Apoptotic photoreceptor cell death in mouse models of retinitis pigmentosa. Proc Natl Acad Sci USA. (1994) 91:974–8. 10.1073/pnas.91.3.974 8302876 PMC521436

[54] Cottet S Schorderet DF. Mechanisms of apoptosis in retinitis pigmentosa. Curr Mol Med. (2009) 9:375–83. 10.2174/156652409787847155 19355918

[55] Arango-Gonzalez B Trifunovic D Sahaboglu A Kranz K Michalakis S Farinelli P. Identification of a common non-apoptotic cell death mechanism in hereditary retinal degeneration. PLoS ONE. (2014) 9:e112142. 10.1371/journal.pone.0112142 25392995 PMC4230983

[56] Hamann Sv Schorderet DF Cottet S. Bax-induced apoptosis in leber's congenital amaurosis: a dual role in rod and cone degeneration. PLoS ONE. (2009) 4:e6616. 10.1371/journal.pone.0006616 19672311 PMC2720534

[57] Vargas A Kim HS Baral E Yu WQ Craft CM Lee EJ. Protective effect of clusterin on rod photoreceptor in rat model of retinitis pigmentosa. PLoS ONE. (2017) 12:e0182389. 10.1371/journal.pone.0182389 28767729 PMC5540409

[58] Viringipurampeer IA Metcalfe AL Bashar AE Sivak O Yanai A Mohammadi Z. NLRP3 inflammasome activation drives bystander cone photoreceptor cell death in a P23H rhodopsin model of retinal degeneration. Hum Mol Genet. (2016) 25:1501–16. 10.1093/hmg/ddw029 27008885 PMC4805309

[59] Kunchithapautham K Rohrer B. Autophagy is one of the multiple mechanisms active in photoreceptor degeneration. Autophagy. (2007) 3:65–66. 10.4161/auto.3431 17102584

[60] Zhou Z Doggett TA Sene A Apte RS Ferguson TA. Autophagy supports survival and phototransduction protein levels in rod photoreceptors. Cell Death Different. (2015) 22:488. 10.1038/cdd.2014.229 25571975 PMC4326583

[61] Moreno ML Merida S Bosch-Morell F Miranda M Villar VM. Autophagy dysfunction and oxidative stress, two related mechanisms implicated in retinitis pigmentosa. Front Physiol. (2018) 9:1008. 10.3389/fphys.2018.01008 30093867 PMC6070619

[62] Yao J Jia L Feathers K Lin C Khan NW Klionsky DJ Ferguson TA Zacks DN. Autophagy-mediated catabolism of visual transduction proteins prevents retinal degeneration. Autophagy. (2016) 12:2439–2450. 10.1080/15548627.2016.1238553 27753525 PMC5173283

[63] Marigo V. Programmed cell death in retinal degeneration: targeting apoptosis in photoreceptors as potential therapy for retinal degeneration. Cell Cycle. (2007) 6:652–655. 10.4161/cc.6.6.4029 17374995

[64] Athanasiou D Aguila M Bellingham J Li W McCulley C Reeves PJ. The molecular and cellular basis of rhodopsin retinitis pigmentosa reveals potential strategies for therapy. Prog Retin Eye Res. (2018) 62:1–23. 10.1016/j.preteyeres.2017.10.002 29042326 PMC5779616

[65] Zhao B Chen W Jiang R Zhang R Wang Y Wang L. Expression profile of IL-1 family cytokines in aqueous humor and sera of patients with HLA-B27 associated anterior uveitis and idiopathic anterior uveitis. Exp Eye Res. (2015) 138:80–6. 10.1016/j.exer.2015.06.018 26116905

[66] Ten Berge JC Fazil Z van den Born I Wolfs RCW Schreurs MWJ Dik WA. Intraocular cytokine profile and autoimmune reactions in retinitis pigmentosa, age-related macular degeneration, glaucoma and cataract. Acta Ophthalmol. (2019) 97:185–92. 10.1111/aos.13899 30298670 PMC6585720

[67] Lu B Yin H Tang Q Wang W Luo C Chen X. Multiple cytokine analyses of aqueous humor from the patients with retinitis pigmentosa. Cytokine. (2020) 127:154943. 10.1016/j.cyto.2019.154943 31810025

[68] Nakamura T Fujisaka Y Tamura Y Tsuji H Matsunaga N Yoshida S. Large cell neuroendocrine carcinoma of the lung with cancer-associated retinopathy. Case Rep Oncol. (2015) 8:153–8. 10.1159/000380943 25873883 PMC4386109

[69] Kyger M Worley A Adamus G. Autoimmune responses against photoreceptor antigens during retinal degeneration and their role in macrophage recruitment into retinas of RCS rats. J Neuroimmunol. (2013) 254:91–100. 10.1016/j.jneuroim.2012.10.007 23110938 PMC3534925

[70] Gorbatyuk M Gorbatyuk O. Review: retinal degeneration: focus on the unfolded protein response. Mol Vis. (2013) 19:1985. 24068865 PMC3782367

[71] Rana T Shinde VM Starr CR Kruglov AA Boitet ER Kotla P. An activated unfolded protein response promotes retinal degeneration and triggers an inflammatory response in the mouse retina. Cell Death Dis. (2014) 5:e1578. 10.1038/cddis.2014.539 25522272 PMC4454166

[72] Murakami Y Ikeda Y Nakatake S Fujiwara K Tachibana T Yoshida N. C-Reactive protein and progression of vision loss in retinitis pigmentosa. Acta Ophthalmol. (2018) 96:e174–9. 10.1111/aos.13502 28636270

[73] Panda S Ding JL. Natural antibodies bridge innate and adaptive immunity. J Immunol. (2015) 194:13–20. 10.4049/jimmunol.1400844 25527792

[74] Zhang Y Garcia-Ibanez L Toellner KM. Regulation of germinal center B-cell differentiation. Immunol Rev. (2016) 270:8–19. 10.1111/imr.12396 26864101 PMC4755139

[75] Nagele EP Han M Acharya NK DeMarshall C Kosciuk MC Nagele RG. Natural IgG autoantibodies are abundant and ubiquitous in human sera, and their number is influenced by age, gender, and disease. PLoS ONE. (2013) 8:e60726. 10.1371/journal.pone.0060726 23589757 PMC3617628

[76] Heckenlively JR Fawzi AA Oversier J Jordan BL Aptsiauri N. Autoimmune retinopathy: patients with antirecoverin immunoreactivity and panretinal degeneration. Arch Ophthalmol. (2000) 118:1525–1533. 10.1001/archopht.118.11.1525 11074809

[77] Adamus G. Autoantibody targets and their cancer relationship in the pathogenicity of paraneoplastic retinopathy. Autoimmun Rev. (2009) 8:410–4. 10.1016/j.autrev.2009.01.002 19168157 PMC2680817

[78] Maeda T Maeda A Maruyama I Ogawa KI Kuroki Y Sahara H. Mechanisms of photoreceptor cell death in cancer-associated retinopathy. Invest Ophthalmol Vis Sci. (2001) 42:705–12. 11222531

[79] Brinkman CJ Pinckers AJ Broekhuyse RM. Immune reactivity to different retinal antigens in patients suffering from retinitis pigmentosa. Invest Ophthalmol Vis Sci. (1980) 19:743–50. 6993414

[80] Heckenlively JR Solish AM Chant SM Meyers-Elliott RH. Autoimmunity in hereditary retinal degenerations. II. Clinical studies: antiretinal antibodies and fluorescein angiogram findings. Br J Ophthalmol. (1985) 69:758–64. 10.1136/bjo.69.10.758 4052361 PMC1040734

[81] Broekhuyse RM van Herck M Pinckers AJ Winkens HJ van Vugt AH Ryckaert S. Immune responsiveness to retinal S-antigen and opsin in serpiginous choroiditis and other retinal diseases. Doc Ophthalmol. (1988) 69:83–93. 10.1007/BF00154420 2971518

[82] Sato T Nishiguchi KM Fujita K Miya F Inoue T Sasaki E. Serum anti-recoverin antibodies is found in elderly patients with retinitis pigmentosa and cancer. Acta Ophthalmol. (2020) 98:e722–9. 10.1111/aos.14373 32043815

[83] Tamm SA Whitcup SM Gery I Wiggert B Nussenblatt RB Kaiser-Kupfer MI. Immune response to retinal antigens in patients with gyrate atrophy and other hereditary retinal dystrophies. Ocul Immunol Inflamm. (2001) 9:75–84. 10.1076/ocii.9.2.75.3972 11449323

[84] Heredia Garcia CD Garcia Calderon PA. Evolution time and longitudinal studies of the anti-S-antigen antibody titers in retinitis pigmentosa. Retina. (1989) 9:237–241. 10.1097/00006982-198909030-00013 2595116

[85] Doekes G Luyendijk L Gerritsen MJ Kijlstra A. Anti-retinal S-antigen antibodies in human sera: a comparison of reactivity in ELISA with human or bovine S-antigen. Int Ophthalmol. (1992) 16:147–152. 10.1007/BF00916433 1452417

[86] Berson EL Rosner B Weigel-DiFranco C Dryja TP Sandberg MA. Disease progression in patients with dominant retinitis pigmentosa and rhodopsin mutations. Invest Ophthalmol Vis Sci. (2002) 43:3027–36. 12202526

[87] Maryam A Vedithi SC Khalid RR Alsulami AF Torres PHM Siddiqi AR. The molecular organization of human cGMP specific phosphodiesterase 6 (PDE6): structural implications of somatic mutations in cancer and retinitis pigmentosa. Comput Struct Biotechnol J. (2019) 17:378–89. 10.1016/j.csbj.2019.03.004 30962868 PMC6434069

[88] Qendro V Bugos GA Lundgren DH Glynn J Han MH Han DK. Integrative proteomics, genomics, and translational immunology approaches reveal mutated forms of Proteolipid Protein 1 (PLP1) and mutant-specific immune response in multiple sclerosis. Proteomics. (2017) 17:322. 10.1002/pmic.201600322 28191734

[89] Plotz PH. The autoantibody repertoire: searching for order. Nat Rev Immunol. (2003) 3:nri976. 10.1038/nri976 12511877

[90] Iannaccone A Radic MZ. Increased protein citrullination as a trigger for resident immune system activation, intraretinal inflammation, and promotion of anti-retinal autoimmunity: intersecting paths in retinal degenerations of potential therapeutic relevance. Adv Exp Med Biol. (2019) 1185:175–9. 10.1007/978-3-030-27378-1_29 31884608

[91] Hollingsworth TJ Hubbard MG Levi HJ White W Wang X Simpson R. Proinflammatory pathways are activated in the human Q344X rhodopsin knock-in mouse model of retinitis pigmentosa. Biomolecules. (2021) 11:1163. 10.3390/biom11081163 34439829 PMC8393353

[92] Stadler M Arnold D Frieden S Luginbuhl S Stadler B. Single nucleotide polymorphisms as a prerequisite for autoantigens. Eur J Immunol. (2005) 35:eji.200425481. 10.1002/eji.200425481 15627977

[93] Oppenheim JJ Dong HF Plotz P Caspi RR Dykstra M Pierce S. Autoantigens act as tissue-specific chemoattractants. J Leukoc Biol. (2005) 77:854–61. 10.1189/jlb.1004623 15917448

[94] Bei R Masuelli L Palumbo C Modesti M Modesti A. A common repertoire of autoantibodies is shared by cancer and autoimmune disease patients: inflammation in their induction and impact on tumor growth. Cancer Lett. (2009) 281:8–23. 10.1016/j.canlet.2008.11.009 19091462

[95] Howard OMZ Dong HF Su SB Caspi RR Chen X Plotz P. Autoantigens signal through chemokine receptors: uveitis antigens induce CXCR3- and CXCR5-expressing lymphocytes and immature dendritic cells to migrate. Blood. (2005) 105:4207–14. 10.1182/blood-2004-07-2697 15713799 PMC1895027

[96] Bachmaier K Krawczyk C Kozieradzki I Kong YY Sasaki T Oliveira-dos-Santos A. Negative regulation of lymphocyte activation and autoimmunity by the molecular adaptor Cbl-b. Nature. (2000) 403:211–6. 10.1038/35003228 10646608

[97] Strong S Liew G Michaelides M. Retinitis pigmentosa-associated cystoid macular oedema: pathogenesis and avenues of intervention. Br J Ophthalmol. (2017) 101:31–7. 10.1136/bjophthalmol-2016-309376 27913439 PMC5256121

[98] Rothova A. Inflammatory cystoid macular edema. Curr Opin Ophthalmol. (2007) 18:487–92. 10.1097/ICU.0b013e3282f03d2e 18163001

[99] Heckenlively JR Jordan BL Aptsiauri N. Association of antiretinal antibodies and cystoid macular edema in patients with retinitis pigmentosa. Am J Ophthalmol. (1999) 127:565–73. 10.1016/S0002-9394(98)00446-2 10334350

[100] Wolfensberger TJ Aptsiauri N Godley B Downes S Bird AC. Antiretinal antibodies associated with cystoid macular edema. Klin Monbl Augenheilkd. (2000) 216:283–5. 10.1055/s-2000-10561 10863693

[101] Grover S Adamus G Fishman GA. Is there an association of anti-retinal antibodies and cystoid macular edema in patients with retinitis pigmentosa? Invest. Ophthalmol. Vis. Sci. (2006) 47:5795.

[102] Mosaad YM. Clinical role of human leukocyte antigen in health and disease. Scand J Immunol. (2015) 82:283–306. 10.1111/sji.12329 26099424

[103] Heckenlively JR Bastek JV Pearlman JT Gladden J Terasaki P. HLA typing in retinitis pigmentosa. Br J Ophthalmol. (1981) 65:131–2. 10.1136/bjo.65.2.131 7459315 PMC1039441

[104] Castagna I Fama F Pettinato G Palamara F Trombetta CJ. HLA typing and retinitis pigmentosa. Ophthalmologica. (1996) 210:152–4. 10.1159/000310696 8738458

[105] Agardh D Gaur LK Agardh E Landin-Olsson M Agardh C-D Lernmark Å. HLA-DQB1*0201/0302 is associated with severe retinopathy in patients with IDDM. Diabetologia. (1996) 39:1313–7. 10.1007/s001250050575 8932997

[106] Holland GN Cornell PJ Park MS Barbetti A Yuge J Kreiger AE. An association between acute retinal necrosis syndrome and HLA-DQw7 and phenotype Bw62,DR4. Am J Ophthalmol. (1989) 108:370–4. 10.1016/S0002-9394(14)73303-3 2801857

[107] Liversidge JM Sewell HF Forrester JV. Human retinal pigment epithelial cells differentially express MHC class II (HLA, DP, DR and DQ) antigens in response to *in vitro* stimulation with lymphokine or purified IFN-gamma. Clin Exp Immunol. (1988) 73:489–94. 3145163 PMC1541780

[108] Caspi RR Chan CC Grubbs BG Silver PB Wiggert B Parsa CF. Endogenous systemic IFN-gamma has a protective role against ocular autoimmunity in mice. J Immunol. (1994) 152:890–9. 8283058

[109] Nagasaka Y Ito Y Ueno S Terasaki H. Number of hyperreflective foci in the outer retina correlates with inflammation and photoreceptor degeneration in retinitis pigmentosa. Ophthalmol Retina. (2018) 2:726–34. 10.1016/j.oret.2017.07.020 31047383

[110] Massengill MT Ahmed CM Lewin AS Ildefonso CJ. Neuroinflammation in Retinitis Pigmentosa, Diabetic Retinopathy, and Age-Related Macular Degeneration: A Minireview. Cham: Springer International Publishing (2018). p. 185–91. 10.1007/978-3-319-75402-4_23 29721943

[111] Forrester JV Xu H Kuffová L Dick AD McMenamin PG. Dendritic cell physiology and function in the eye. Immunol Rev. (2010) 234:282–304. 10.1111/j.0105-2896.2009.00873.x 20193026

[112] Xu H Chen M Forrester JV. Para-inflammation in the aging retina. Progr Retinal Eye Res. (2009) 28:348–68. 10.1016/j.preteyeres.2009.06.001 19560552

[113] Chen M Xu H. Parainflammation, chronic inflammation, and age-related macular degeneration. J Leukocyte Biol. (2015) 98:713–25. 10.1189/jlb.3RI0615-239R 26292978 PMC4733662

[114] Chinnery HR McMenamin PG Dando SJ. Macrophage physiology in the eye. Pflügers Archiv. (2017) 5:1–15. 10.1007/s00424-017-1947-5 28233124

[115] Hisatomi T Sakamoto T Sonoda K-h Tsutsumi C Qiao H Enaida H. Clearance of apoptotic photoreceptors. Am J Pathol. (2003) 162:1869–79. 10.1016/S0002-9440(10)64321-0 12759244 PMC1868143

[116] Karlstetter M Scholz R Rutar M Wong WT Provis JM Langmann T. Retinal microglia: just bystander or target for therapy? Prog Retin Eye Res. (2015) 45:4. 10.1016/j.preteyeres.2014.11.004 25476242

[117] Blank T Goldmann T Koch M Amann L Schön C Bonin M. Early microglia activation precedes photoreceptor degeneration in a mouse model of CNGB1-linked retinitis pigmentosa. Front Immunol. (2017) 8:1930. 10.3389/fimmu.2017.01930 29354133 PMC5760536

[118] Langmann T. Microglia activation in retinal degeneration. J Leukoc Biol. (2007) 81:1345–51. 10.1189/jlb.0207114 17405851

[119] Rashid K Akhtar-Schaefer I Langmann T. Microglia in retinal degeneration. Front Immunol. (2019) 10:1975. 10.3389/fimmu.2019.01975 31481963 PMC6710350

[120] Sivakumar V Foulds WS Luu CD Ling E-A Kaur C. Retinal ganglion cell death is induced by microglia derived pro-inflammatory cytokines in the hypoxic neonatal retina. J Pathol. (2011) 224:245–60. 10.1002/path.2858 21404274

[121] Kohno H Chen Y Kevany BM Pearlman E Miyagi M Maeda T. Photoreceptor proteins initiate microglial activation *via* toll-like receptor 4 in retinal degeneration mediated by all-trans-retinal. J Biol Chem. (2013) 288:15326–41. 10.1074/jbc.M112.448712 23572532 PMC3663552

[122] Xu H Chen M. Targeting the complement system for the management of retinal inflammatory and degenerative diseases. Eur J Pharmacol. (2016) 787:94–104. 10.1016/j.ejphar.2016.03.001 26948311 PMC5026403

[123] Silverman SM Ma W Wang X Zhao L Wong WT. C3- and CR3-dependent microglial clearance protects photoreceptors in retinitis pigmentosa. J Exp Med. (2019) 216:1925–43. 10.1084/jem.20190009 31209071 PMC6683998

[124] Uren PJ Lee JT Doroudchi MM Smith AD Horsager A. A profile of transcriptomic changes in the rd10 mouse model of retinitis pigmentosa. Mol Vis. (2014) 20:1612–28. 25489233 PMC4235044

[125] Rohrer B Demos C Frigg R Grimm C. Classical complement activation and acquired immune response pathways are not essential for retinal degeneration in the rd1 mouse. Exp Eye Res. (2007) 84:82–91. 10.1016/j.exer.2006.08.017 17069800 PMC1885545

[126] Humphries MM Kenna PF Campbell M Tam LCS Nguyen ATH Farrar GJ. C1q enhances cone photoreceptor survival in a mouse model of autosomal recessive retinitis pigmentosa. Eur J Hum Genet. (2012) 20:64–8. 10.1038/ejhg.2011.151 21863053 PMC3234518

[127] Anderson DH Radeke MJ Gallo NB Chapin EA Johnson PT Curletti CR. The pivotal role of the complement system in aging and age-related macular degeneration: hypothesis re-visited. Prog Retin Eye Res. (2010) 29:95–112. 10.1016/j.preteyeres.2009.11.003 19961953 PMC3641842

[128] Radu RA Hu J Yuan Q Welch DL Makshanoff J Lloyd M. Complement system dysregulation and inflammation in the retinal pigment epithelium of a mouse model for Stargardt macular degeneration. J Biol Chem. (2011) 286:18593–601. 10.1074/jbc.M110.191866 21464132 PMC3099675

[129] Yang L Kim J-H Kovacs KD Arroyo JG Chen DF. Minocycline inhibition of photoreceptor degeneration. Arch Ophthalmol. (2009) 127:1475–80. 10.1001/archophthalmol.2009.288 19901213

